# Differentiation and transplantation of human induced pluripotent stem cell-derived otic epithelial progenitors in mouse cochlea

**DOI:** 10.1186/s13287-018-0967-1

**Published:** 2018-08-29

**Authors:** Jianling Chen, Fanfan Hong, Cui Zhang, Liang Li, Cuicui Wang, Haosong Shi, Yong Fu, Jinfu Wang

**Affiliations:** 1 Institute of Cell and Development, College of Life Sciences, Zi-Jin-Gang Campus of Zhejiang University, Room 307, No.866, Yuhangtang Road, Hangzhou, 310058 Zhejiang China; 2 Department of Otorhinolaryngology, the Sixth People’s Hospital, School of Medicine, Shanghai Jiaotong University, Shanghai, China; 3 Department of ENT, Head and Neck Surgery, the Children’s Hospital, Zhejiang University School of Medicine, Zhejiang, China; 4 Department of Otolaryngology, the Children Hospital, School of Medicine, Bin-Jiang Campus of Zhejiang University, No. 3333, Binsheng Road, Hangzhou, 310051 Zhejiang China

**Keywords:** Sensorineural hearing loss, Induced pluripotent stem cells, Hair cell-like cells, Otic epithelial progenitors, Synaptic connection

## Abstract

**Background:**

Inner ear hair cells as mechanoreceptors are extremely important for hearing. Defects in hair cells are a major cause of deafness. Induced pluripotent stem cells (iPSCs) are promising for regenerating inner ear hair cells and treating hearing loss. Here, we investigated migration, differentiation, and synaptic connections of transplanted otic epithelial progenitors (OEPs) derived from human iPSCs in mouse cochlea.

**Methods:**

Human urinary cells isolated from a healthy donor were reprogramed to form iPSCs that were induced to differentiate into OEPs and hair cell-like cells. Immunocytochemistry, electrophysiological examination, and scanning electron microscopy were used to examine characteristics of induced hair cell-like cells. OEP-derived hair cell-like cells were cocultured with spiral ganglion neurons (SGNs), and the markers of synaptic connections were detected using immunocytochemistry and transmission electron microscope. In vivo, OEPs derived from iPSCs were transplanted into the cochlea of mice by injection through the round window. Migration, differentiation, and synaptic connections of transplanted cells were also examined by thin cochlear sectioning and immunohistochemistry.

**Results:**

The induced hair cell-like cells displayed typical morphological characteristics and electrophysiological properties specific to inner hair cells. In vitro, OEP-derived hair cell-like cells formed synaptic connections with SGNs in coculture. In vivo, some of the transplanted cells migrated to the site of the resident hair cells in the organ of Corti, differentiated into hair cell-like cells, and formed synaptic connections with native SGNs.

**Conclusions:**

We conclude that the transplantation of OEPs is feasible for the regeneration of hair cells. These results present a substantial reference for a cell-based therapy for the loss of hair cells.

## Background

Hearing loss is an important sensory impairment in humans. It causes significant health burden on society since it impedes speech and language development and decreases the quality of life. Sensorineural hearing loss (SNHL) results from loss or damage to the hair cells (the sensorineural element) in the inner ear. It is a common form of hearing impairment worldwide, affecting millions of people. Mammalian sensory hair cells fail to regenerate spontaneously when injured by several causes including noise, ototoxic drugs, aging, and genetic factors [1, 2]. Currently, limited treatment is available to rescue hearing loss and consists of prosthetic devices such as hearing aids and cochlear implants. Thus, there is a need to develop new therapeutic methods to aid millions of people suffering from hearing impairment. Advances in somatic cell programing technology offer a glimmer of hope for developing new therapies to treat hearing loss. Induced pluripotent stem cells (iPSCs) are generated from somatic cells including skin fibroblasts [3, 4], cord blood [5], keratinocytes [6], lymphocytes [7], and urinary cells [8, 9]. Recent studies reveal induced differentiation of iPSCs into hair cell-like cells with morphological and electrophysiological characteristics typical of hair cells [10, 11], demonstrating the possibility of regenerating hair cells.

In the present study, urinary cells isolated from a healthy donor were reprogrammed into iPSCs. iPSCs were induced to differentiate into otic epithelial progenitors (OEPs), which further differentiated into hair cell-like cells. Hair cell-like cells were then cocultured with spiral ganglion neurons (SGNs) to analyze their potential to form synaptic connections with SGNs. Finally, iPSC-derived OEPs were transplanted into the cochlea of mice to examine the engrafting and differentiation of transplanted cells committed to form hair cells in the organ of Corti.

## Methods

### Collection of urinary cells and generation of iPSCs

After obtaining written informed consent from the donor, urine sample was collected from a healthy female aged 26 years following approval by the Zhejiang Health Bureau located in Hangzhou. A total volume of 200 mL urine was collected, dispersed into 50-mL tubes, and centrifuged at 400×*g* for 10 min at room temperature (20–25 °C). The supernatant was discarded leaving approximately 1 mL urine in the tube. The residual urine sample (1 mL) from each tube was pooled into a single tube and 10 mL phosphate-buffered saline (PBS) containing 2.5 μg/mL amphotericin B (Amresco, Shanghai, China), 100 U/mL penicillin, and 100 μg/mL streptomycin (Gibco, Shanghai, China) was added and centrifuged at 400×*g* for 10 min. The supernatant was discarded. The residual 0.2 mL sample was resuspended in 1 mL primary medium (Dulbecco’s modified Eagle’s medium/Nutrient Mixture Ham’s F-12 (DMEM/F12; 1:1; Gibco) supplemented with 10% (v/v) fetal bovine serum (FBS; Gibco), SingleQuot Kit CC-4127 renal epithelial cell growth medium (REGM; Lonza, Shanghai, China), 2.5 μg/mL amphotericin B, 100 U/mL penicillin, and 100 μg/mL streptomycin (Gibco)) and cultured at 37 °C, 5% CO_2_, and 95% humidity. On the first and second day of culture, 500 μL primary medium was added to the cells. Later, half the medium was replaced with RE proliferation medium (renal epithelial basal medium (REBM; Lonza) supplemented with SingleQuot Kit CC-4127 REGM). The first complete media change with RE proliferation medium was made after the first cells/colonies were visualized. Subsequently, the culture medium was completely replaced every second day. When most of colonies were grown to confluence, cells were split and seeded in a 12-well plate aided by TryLE™ Express (Gibco). Cells from passage 3 were used for the induction of iPSCs.

iPSCs were generated from urinary cells using a retroviral transduction method with the four Yamanaka factors (OCT4, SOX2, c-MYC, and KLF4) as previously described [12] with a few modifications. HEK293T cells used in the study were gifted by Prof. Guan (Zhejiang University School of Medicine, China). Briefly, HEK293T cells seeded at a density of 1.2 × 10^6^ cells/well in 0.1% (w/v) gelatin (Sigma, Shanghai, China)-coated six-well plates were cultured in HEK293T medium (DMEM/high glucose (Gibco) supplemented with 10% FBS, 1% (v/v) GlutaMAX, and 1% (v/v) sodium pyruvate (Gibco)). When HEK293T cells reached 80% confluence, they were transfected with 3.3 μg pCL-ECO packaging vector combined with 3.3 μg each of PMX-GFP, PMX-OCT4, PMX-SOX2, PMX-KLF4, and PMX-c-MYC (gifted by Prof. Guan) using Lipofectamine™ 2000 (Invitrogen, Shanghai, China) in six-well plates. PMX-GFP was used to determine the transfection efficiency. At 6 h post-transfection, the culture medium was replaced with 2 mL fresh HEK293T medium supplemented with sodium butyrate (10 mM; Sigma). After 12 h of culture, the medium was again replaced with 2 mL fresh HEK293T medium. At 48 h post-transfection, virus-containing supernatants were collected for use in first infection and 2 mL fresh HEK293T medium was added to each well for further retroviral production. Viral supernatants containing the four Yamanaka factors were mixed and filtered through a 0.45-μm syringe filter. The resultant viral supernatant was mixed with 750 μL RE proliferation medium and an equal volume of MC proliferation medium (REBM supplemented with 10% (v/v) FBS, 1% (v/v) GlutaMAX, 1% (v/v) nonessential amino acids (NEAA), epidermal growth factor (EGF; 5 ng/mL), basic fibroblast growth factor (bFGF; 5 ng/mL; R&D, Shanghai, China), 100 U/mL penicillin, and 100 μg/mL streptomycin). Green fluorescent protein (GFP) containing viral supernatant was mixed with 500 μL each of RE and MC proliferation medium. Viral supernatants containing polybrene (10 μg/mL; Sigma) were used to infect urinary cells. Before the first infection, urinary cells were seeded at a density of 6 × 10^4^ cells/well in six-well plates and cultured overnight in RE proliferation medium. At 12 h postinfection the medium was replaced with 1 mL RE and an equal volume of MC proliferation medium. At 72 h post-transfection, the viral supernatant produced by HEK293T was collected again and used for the second infection. At 12 h following the second infection, the medium was replaced with 1 mL RE proliferation medium and an equal volume of MC proliferation medium supplemented with vitamin C (VC; 50 μg/ mL; Sigma). Thereafter, the medium was replaced with fresh medium every day. At 4–5 days following the second infection, mouse embryonic fibroblast (MEF) cells treated with mitomycin C (10 ng/mL; Sigma) were seeded at a density of 1.5 × 10^6^ cells in a 10-cm gelatin-coated dish as feeder cells. Infected urinary cells stabilized relative to their visible morphological changes were trypsinized with 0.25% trypsin-ethylenediaminetetraacetic acid (EDTA) (Gibco). The trypsinized cells were resuspended in 10 mL medium containing 5 mL each of human embryonic stem cell (hESC) medium (DMEM/F12 (1:1) supplemented with 20% (v/v) knockout serum replacement (KOSR), 1% GlutaMAX, 1% NEAA, 100 μM β-mercaptoethanol (Gibco), and bFGF (10 ng/mL)) and dFBS medium (DMEM/high glucose supplemented with 20% FBS, 1% GlutaMAX, 1% NEAA, 100 μM β-mercaptoethanol, and 10 ng/mL bFGF) supplemented with VC (50 μg/mL) and valproic acid (VPA; 1 mM; Sigma), and were seeded on feeder cells for iPSC induction. Until 7 days following iPSC induction the medium was daily replaced with fresh medium. At 8 days following iPSC induction the medium was replaced without VPA. By 10 days following iPSC induction the medium was replaced with fresh mTesR1 medium. Seven days after the appearance of hESC-like colonies, the colonies were harvested, transferred onto new feeder cells, and cultured in hESC medium for passaging. Alkaline phosphatase staining was performed using a leukocyte alkaline phosphatase kit (Sigma) according to the manufacturer’s instructions. The formation of embryoid bodies (EBs) and teratomas by iPSCs were assayed to characterize the totipotency of induced iPSCs as previously described [8, 9, 13]. To prepare the cells expressing GFP used for the transplantation experiment, 2.5 μg pGIP2-EFG, 1.875 μg psPAX2, and 0.625 μg PMD2.G (gifted by Prof. Guan) were transfected into HEK293T cells to produce lentivirus. Then, iPSCs were seed at a density of 1 × 10^5^ cells/well in a six-well plate and infected with the lentivirus as the induction of iPSCs.

### Generation of otic progenitors, hair cell-like cells, and SGN-like cells from iPSCs

For the induction of otic progenitors, iPSCs were treated with 1 mg/mL collagenase IV (Gibco) at 37 °C for 1 h. The cells were harvested, resuspended in 5 mL DMEM/F12 (1:1), and centrifuged at 200×*g* for 1 min at room temperature (20–25 °C). The supernatants were discarded and the pelleted iPSCs were incubated with 0.025% trypsin-EDTA at 37 °C for 0.5 min. Trypsinization was terminated by the addition of trypsin inhibitor (Invitrogen). DMEM/F12 (1:1) medium was added and the cells were mechanically dissociated by pipetting up and down several times. Cells in suspension were passed through a 70-μm cell strainer. iPSCs were seeded at a density of 1.8 × 10^5^ cells/well onto a laminin-coated (5 μg/cm^2^; Invitrogen) 24-well plate with otic progenitor-induced medium (DMEM/F12 (1:1) supplemented with 1% (v/v) N2, 0.5% (v/v) B27 (Gibco), FGF3 (50 ng/mL), and FGF10 (50 ng/mL; R&D)) and incubated at 37 °C, 5% CO_2_, and 95% humidity for 12 days. Thereafter, the medium was replaced with fresh medium every day. After 12 days of induction, the induced cells were examined for the presence of otic progenitors and were characterized by reverse transcription-polymerase chain reaction (RT-PCR) and immunohistochemistry assays.

In the present study, a stepwise digestion method was used to separate otic epithelial progenitors (OEPs) from otic neural progenitors (ONPs) [9]. Otic progenitor colonies were treated with 0.025% trypsin-EDTA at 37 °C for 20 s and observed for partially lifted cells surrounding the epithelial colonies (OEPs). Once colony edges started to curl, the activity of trypsin was terminated by the addition of trypsin inhibitor, and the lifted cells were harvested as ONPs and transferred to a tube containing DMEM/F12 (1:1). The above-mentioned quick trypsinization step was repeated several times until most of the ONPs were separated from OEPs. Later, to collect the cells that remained attached (OEPs), cells were incubated with 0.025% trypsin-EDTA at 37 °C for 40 s, and trypsinization was terminated by the addition of trypsin inhibitor. The dissociated OEPs were collected in another tube containing DMEM/F12 (1:1). The enriched OEPs were transferred onto mitomycin-treated chicken embryonic utricle stromal cells and cultured with hair cell-induced medium (DMEM/F12 (1:1) supplemented with 1% (v/v) N2, 0.5% (v/v) B27, all-*trans* retinoic acid (10^−6^ M; Sigma), and EGF (20 ng/mL)) at 37 °C, 5% CO_2_, and 95% humidity for 3 weeks. The culture medium was replaced every second day. After 3 weeks of induction, the induced cells were examined for the expression of characteristics specific for hair cells by RT-PCR, immunohistochemistry, scanning electron microscopy, and electrophysiology assays.

Enriched ONPs were seeded at a density of 4 × 10^5^ cells/well onto a laminin-coated 24-well plate and incubated with sensory neuron-induced medium (DMEM/F12 (1:1) supplemented with 1% (v/v) N2, 0.5% (v/v) B27, bFGF (20 ng/mL), and sonic hedgehog (Shh-C24II; 500 ng/mL; R&D)). On the third day of induction, the sensory neuron-induced medium was supplemented with neurotrophin 3 (NT3; 10 ng/mL) and brain-derived neurotrophic factor (BDNF; 10 ng/mL; R&D). At 5 days of induction, the medium was replaced without Shh-C24II and, thereafter, the culture medium was replaced completely every second day. After 2 weeks of induction, the induced cells were examined for the expression of characteristics specific for SGNs by RT-PCR and immunohistochemistry assays.

### Establishment of synaptic connections

All animal experiments were approved by the Institutional Animal Care and Use Committee and the Laboratory Animal Welfare Ethical Committee of Zhejiang University. SGNs were isolated from the modiolus of neonatal ICR mice and cultured with DMEM/high glucose supplemented with NT3 (10 ng/mL), BDNF (10 ng/mL), 10% FBS, 1% NEAA, 100 U/mL penicillin, and 100 μg/mL streptomycin. The medium was replaced every day. After 7 days of culture, SGNs were dissociated with 0.025% trypsin-EDTA and seeded at a density of 4 × 10^3^ cells/well along with 1 × 10^4^ cells/well of hair cell-like cells (cultured with hair cell-induced medium for 2 weeks) in a 24-well plate and cocultured with the coculture medium (DMEM/F12 (1:1) supplemented with 1% (v/v) N2, 0.5% (v/v) B27, all-*trans* retinoic acid (10^−6^ M), EGF (20 ng/mL), NT3 (10 ng/mL), and BDNF (10 ng/mL)) at 37 °C, 5% CO_2_, and 95% humidity. The culture medium was replaced every second day until day 7 of coculture.

Furthermore, we examined the synaptic connection between the induced hair cell-like cells and the induced SGN-like cells. ONPs obtained from otic progenitors were cultured with sensory neuron-induced medium for 7 days. The induced SGN-like cells were detached with accutase (Sigma). Cells induced from OEPs (cultured with hair cell-induced medium for 2 weeks) were seeded at a density of 1 × 10^4^ cells/well together with 5 × 10^3^ cells/well of cells induced from ONPs (cultured with sensory neuron-induced medium for 7 days) onto a laminin-coated 24-well plate and cultured in coculture medium at 37 °C, 5% CO_2_, and 95% humidity for 7 days. The culture medium was replaced every second day.

### Transplantation and cochlea processing

In the present study, transplantation experiments were performed on *Slc26a4*-null mice (gifted by Prof. Gao, Shandong University, China). Mutation in *Slc26a4* results in abnormal pendrin protein that causes a defect in hair cells and eventually leads to hearing loss. To help ensure the safety of transplantation, NOD/SCID mice were subcutaneously/intramuscularly injected with OEPs at a density of 1 × 10^7^ cells. Correspondingly equal numbers of iPSCs were injected as a control to detect the presence, if any, of a tumorigenic capacity of transplanted cells in vivo.

Before transplantation, *Slc26a4*-null mice were anesthetized with 4% (w/v) chloral hydrate (Sigma). Under sterile conditions, the otic bulla of the left ear was exposed by a modified retro-auricular approach [14]. Briefly, a small hole was opened on the surface of the otic bulla using a 1-mL syringe needle revealing the round window. The round window membrane was punctured with a 27-G needle. Four microliters (4–5 × 10^5^ cells) of OEPs derived from iPSCs expressing GFP were injected into the scala tympani at a speed of 1 μL/min using a microsyringe pump (WPI, Shanghai, China). The needle was left in place for an additional 1 min and then carefully removed. The hole in the round window was sealed with muscle and the incision was sutured. To prevent rejection, mice were given daily injections (intraperitoneally) of cyclosporine A (500 mg/kg/day) for 3 days.

### Gene expression analysis

Isolated total RNA (1 μg) was reverse transcribed using the RevertAid first strand cDNA synthesis Kit (Thermo Scientific, Beijing, China). Primers were designed using Primer Premier 6.0 Demo and Oligo 7.36 Demo software as shown in Table 1. The PCR profile was as follows: 95 °C for 5 min, followed by 30 cycles at 94 °C for 30 s, primer annealing at a specific temperature as indicated in Table 1 for 30 s, and 72 °C for 1 min. PCR products were electrophoresed on a 1.5% agarose gel and stained with ethidium bromide. Stained PCR amplified bands were visualized under ultraviolet (UV) light and photographed. The results of RT-PCR were confirmed by at least three independent experiments. The housekeeping gene glyceraldehyde-3-phosphate dehydrogenase (GAPDH) was used as an internal standard for RT-PCR.

**Table 1:** Primer sequences for reverse transcription-polymerase chain reaction

Gene name	Forward primer (5’–3’)	Reverse primer (5’–3’)	Amplicon length	Annealing temperature
*OCT4*	GACAGGGGGAGGGGAGGAGCTAGG	CCTCCCTCCAACCAGTTGCCCCAAAC	144 bp	60 °C
*SOX2*	GGGAAATGGAGGGGTGCAAAAGAGG	TTGCGTGAGTGTGGA TGGGATTGGTG	151 bp	62.5 °C
*KLF4*	GAGGGAAGACCAGAATTCCCTTGA	AGAAC AA ACTCACCAAGCACCA	180 bp	60 °C
c-*MYC*	TGCACTGGAACTTACAA ACCCGA	TAA GCA GCT GCA AGG AGA GCC TTT	133 bp	60 °C
*AFP*	GAATGCTGCAAACTGACCACGCTGGAAC	TGGCATTCA AGAGGGTTTTCAGTTGGA	281 bp	62 °C
*CK8*	CCTGGA AGGGCTGACCGACGAGATCAA	CTTCCCAGCCAGGCCTGCAGCTCC	247 bp	65 °C
*CK18*	AGCTCAACGGGATCCTGCTG ACCTTG	CACTATCCGGCGG GTGGTGGTCTTTTG	233 bp	65 °C
*MSX1*	CGAGAGGACCCCGTGGATGCAGAG	GGCGGCCATCTTCAGCTTCT CAG	307 bp	63 °C
*BRACHYURY*	GCCCTCTCCCTCCCCTCCACGCACAG	CGGCGCCGTTGCTCACAGACCACAGG	274 bp	65 °C
*PAX6*	ACCCATTATCCAGATGTGTTTGCCCGAG	ATGGTGAAGCTGGGCATAGGCGG AG	317 bp	62 °C
*SOX 1*	CAACCAGGACCGGGCAAAC	CCTCGGACATGACCTTC AC	146 bp	58 °C
*GAPDH*	GAAGGTCGGAGTCAACGG	GGAAGATGGTGATGGGATT	496 bp	53 °C
*SIX1*	GACTCCGGTTTTCGCCTTTG	TAGTTTGAGCTCCTGGCGTG	878 bp	57 °C
*PAX2*	GAGCGAGTTCTCCGGCAAC	GTCAGACGGGGACGATGTG	225 bp	60 °C
*PAX8*	ACCCCCAAGGTGGTGGAGAAGA	CTCGAGGTGGTGCTGGCTGAAG	450 bp	62 °C
*DLX5*	TTCCAAGCTCCGTTCCAGAC	GTAATGCGGCCAGCTGAAAG	409 bp	57 °C
*EYA1*	TCAGATGCTATCTGCCGCTG	GTGCCATTGGGAGTCATGGA	595 bp	57 °C
*ATOH1*	GCCGCCCAGTATTTGCTACA	GCTAGCCGTCTCTGCTTCTG	260 bp	57 °C
*BRN3C*	TGCAAGAACCCAAATTCTCC	GAGCTCTGGCTTGCTGTTCT	758 bp	55 °C
*MYO7A*	CACATCTTTGCCATTGCTGAC	AGAAGAGAACCTCACAGGCAT	649 bp	55 °C
*ESPIN*	CAGGCATGTCCTCACCCAAT	CGTGGCGGAGTTTGTTCTTG	384 bp	55 °C
*NEUROD1*	GCCCCAGGGTTATGAGACTATCACT	CCGACAGAGCCCAGATGTAGTTCTT	523 bp	59 °C
*POU4F1*	AACAGCAAGCAGCCTCACTT	TTCATCGTGTGGTACGTGGC	245 bp	55 °C
*NTRK2*	AATTGGGTTGGAGCAGGAGC	GGGTCCATGCCACCTTATCC	522 bp	56 °C
*ISLET1*	CAACAAACAAAACGCAAAAC	AAGTCAAACACAATCCCGA	542 bp	50 °C

### Preparation of thin cochlear sections, immunohistochemistry, scanning electron microscopy (SEM), and transmission electron microscopy (TEM)

For preparing thin cochlear sections, cochlea dissected from mice were fixed overnight in 4% paraformaldehyde at 4 °C, decalcified in 10% (w/v) EDTA for 4 days, and placed in 25% sucrose (in PBS) for 1 day. Cochlea were transferred to optimal cutting temperature (OCT; Tissue-Tek) medium and placed under gentle vacuum for 15 min. Cochlea were then frozen at −20 °C for 1 h and thin-sectioned on a cryostat microtome. Thin sections (15 μm thick) were mounted on slides for immunohistochemistry analysis. For the preparation of the cochlear basement membrane, the cochlea was fixed and decalcified as stated above. Temporal bone and tectorial membrane were carefully removed using fine tweezers. The basement membrane was separated from the modiolus, spiral ligament, and the spiral ganglion present adjacent to it. The isolated basement membrane was dissected for whole-mount immunofluorescence.

For immunohistochemistry assay, cells and tissue sections were fixed overnight in 4% paraformaldehyde at 4 °C, permeabilized with 0.25% (v/v) Triton X-100 for 30 min, blocked in 6% (w/v) bovine serum albumin for 1 h at room temperature (20–25 °C), and incubated overnight with primary antibodies at room temperature (20–25 °C). Primary antibodies used in the present study are anti-Oct4 (1:350, rabbit polyclonal), anti-Nanog (1:1000, rabbit polyclonal), anti-SSEA (1:70, mouse monoclonal), anti-TRA-1-81 (1:100, mouse monoclonal), anti-TRA-1-60 (1:100, mouse monoclonal), anti-PAX8 (1:200, mouse monoclonal), anti-PAX2 (1:250, rabbit polyclonal), anti-NESTIN (1:250, rabbit polyclonal), anti-SOX2 (1:900, rabbit polyclonal), anti-MYO7A (1:500, rabbit monoclonal), anti-ATOH1 (Atonal homolog1; 1:1000, rabbit polyclonal), anti-BRN3C (1:50, mouse monoclonal), anti-β-tubulin III (TUJ-1; 1:1000, mouse monoclonal), anti-NF200 (1:500, mouse monoclonal), anti-BRN3A (1:500, rabbit polyclonal; Millipore), anti-synaptophysin (SYP; 1:50, mouse monoclonal), anti-CtBP2 (1:400, goat polyclonal; Santa Cruz), anti-GFP (1:1000, goat polyclonal), and anti-GluR2 (1:250, mouse monoclonal). All antibodies (where not otherwise specified) were purchased from Abcam. Cells and tissue sections were then rinsed three times with PBS before incubation with the secondary antibody for 1 h at room temperature (20–25 °C). Alexa Fluor secondary antibodies (Jackson ImmunoResearch, Shanghai, China) used in the present study included donkey anti-rabbit Alex Fluor 594, donkey anti-goat Alex Fluor 405 or Alex Fluor a-488, and donkey anti-mouse Alex Fluor 488 or Alex Fluor 647 (1:400 dilution). Nuclei were counterstained with 4′,6-diamidino-2-phenylindole (DAPI; Sigma). Confocal images were taken using the Carl Zeiss LSM-510 microscope.

For SEM, cells and tissue sections were fixed overnight in 2.5% glutaraldehyde at 4 °C, washed with PBS, and postfixed in 1% osmic acid for 1.5 h. Cells and tissue sections were dehydrated in an ethanol gradient (30%, 50%, 70%, 90%, and 95%; 15 min each) followed by absolute ethanol, twice for 10 min each. The samples were dried by critical point drying for 2 h, plated with gold, and observed on a metal conductive column for SEM. TEM was performed as previously described [15].

### Electrophysiology

Membrane currents of 3-week-old cultured cells under hair cell differentiation conditions were measured using the whole-cell patch-clamp technique with the aid of an amplifier (EPC 10; HEKA, Lambrecht/Pfalz). Recordings were performed at room temperature (20–25 °C). The extracellular solution (pH 7.3–7.5) contained (in mM): 135 NaCl, 0.9 MgCl_2_, 5.8 KCl, 0.7 NaH_2_PO_4,_ 1.3 CaCl_2_, 5.6 d-glucose, 10 HEPES-NaOH, and 2 sodium pyruvate. Cells were flooded with the extracellular solution and visualized using the phase-contrast mode of an inverted microscope (TE-2000 U; Nikon, Tokyo, Japan). Patch electrodes with a resistance of 4–8 MΩ were pulled from the borosilicate capillary glass using a vertical pipette puller (P-97; Narishige). Inward Ca^2+^ current (*I*_Ca_) was recorded using the pipette filling solution (intracellular fluid), containing (in mM): 131 CsCl, 3 MgCl_2_, 1 EGTA-KOH, 5 Na_2_ATP, 5 HEPES-KOH, and 10 sodium phosphocreatine (pH 7.3). Outward K^+^ current (*I*_K1_) and inward K^+^ current (*I*_K_) values were recorded using the pipette filling solution (pH 7.3) containing (in mM): 131 KCl, 3 MgCl_2_, 1 EGTA-KOH, 5 Na_2_ATP, 5 HEPES-KOH, and 10 sodium phosphocreatine. All reagents were purchased from Sigma.

Voltage protocol application and data acquisition were performed using the Pulse 6.0 software (HEKA). Recordings were filtered at 1–3 kHz, sampled at 3–10 kHz, and stored on computer for off-line analysis using the Clamfit and Origin software (OriginLab). Capacitance and series resistance were corrected. The series resistance compensation was 80–94%. The peak current (pA) that developed during the depolarization step was considered the maximum current. Membrane capacitance (pF) was recorded for analyzing the current density. Membrane currents were elicited by applying voltage steps in 10-mV nominal increments or decrements from the holding potential of −84 mV (for *I*_K_ and *I*_Ca_) or −64 mV (for *I*_K1_).

### Statistical analysis

Data are represented as the mean ± standard deviation (SD) of independent experiments (*n* = independent biological replicates).

## Results

### Generation and characterization of iPSCs

**Fig. 1:**
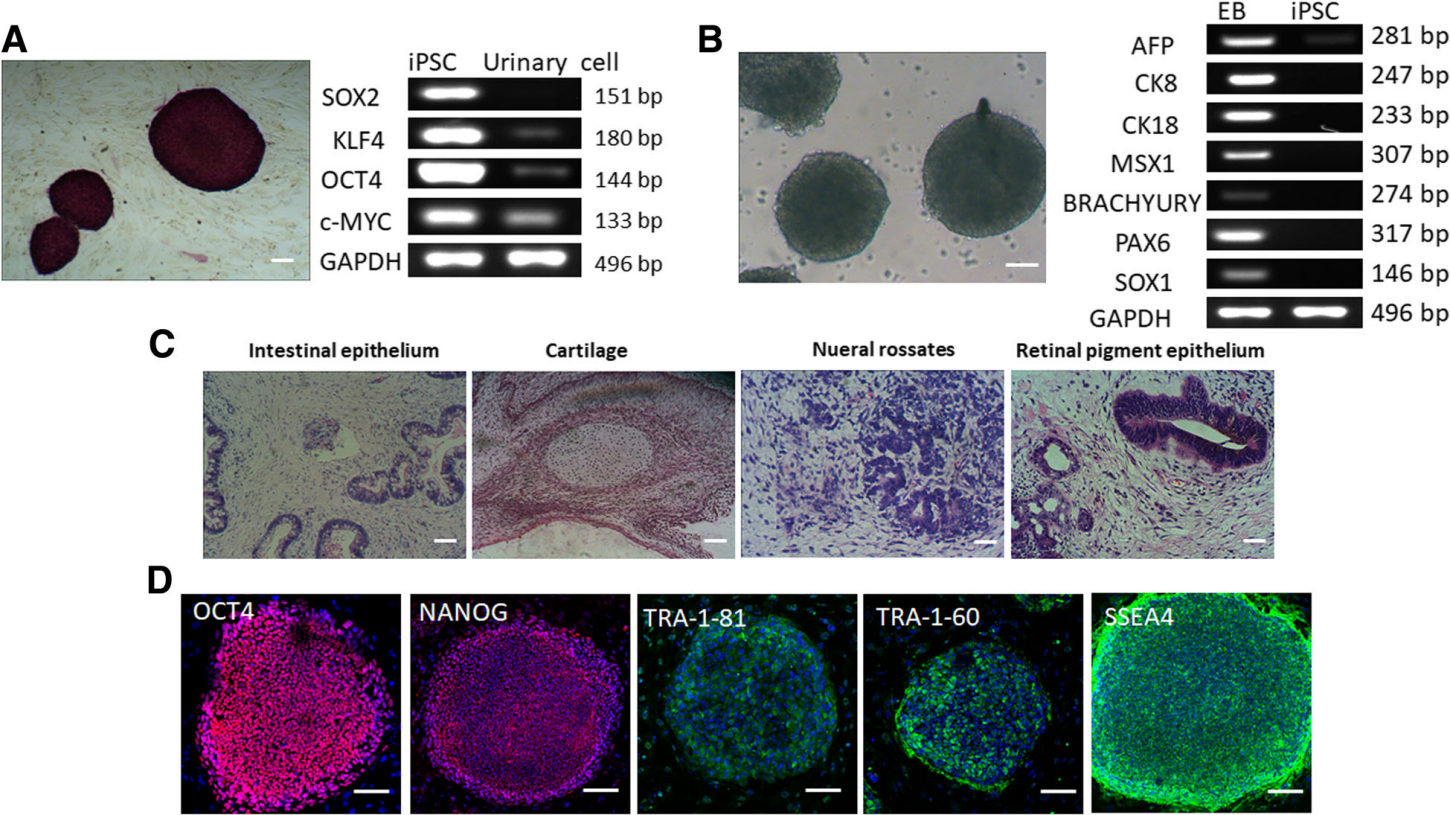
Identification of induced pluripotent stem cells (iPSCs) formed from urinary cells. **a** Alkaline phosphatase staining of iPSCs. Endogenous expression of SOX2, KLF, OCT4, and c-MYC in iPSCs compared with that in urinary cells. Scale bars = 100 μm. **b** Formation of embryoid bodies (EBs) in suspension culture of iPSCs. EBs expressed AFP, CK8, and CK18 specific for endoderm, MSX1 and Brachyury specific for mesoderm, and PAX6 and SOX1 specific for ectoderm. Scale bars = 100 μm. **c** Hematoxylin and eosin staining of teratoma derived from iPSCs in NOD/SCID mouse displaying various structures, including gut epithelium in endoderm, neural rosettes and retinal pigment in ectoderm, and cartilage in mesoderm. **d** Immunostaining for pluripotent markers OCT4, NANOG, TRA-1-81, TRA-1-60, and SSEA4 in iPSCs. Nuclei were stained with 4’,6-diamidino-2-phenylindole (DAPI; blue). Scale bars = 200 μm

Urinary cells isolated from the urine sample of a normal donor were infected with four retroviruses carrying the transcription factor genes OCT4, SOX2, KLF4, and c-MYC. ESC-like colonies were formed with clear edges at 20 days postinfection. Colonies were harvested and cultured on mitomycin-treated MEFs. Analysis of totipotency, a characteristic feature of iPSCs, was used for their identification. Our results showed that ESC-like colonies were positive for alkaline phosphatase. RT-PCR analysis confirmed enhanced expression of the endogenous pluripotent genes SOX2, KLF4, OCT4, and c-MYC in ESC-like colonies compared with that in urinary cells, indicating that transduction of exogenous genes could activate the expression of endogenous pluripotent genes (Fig. 1a). Exposure of ESC-like colonies to EB conditions for 8 days resulted in the expression of AFP, CK8, and CK18 specific for endoderm, MSX1 and Brachyury specific for mesoderm, and PAX6 and SOX1 specific for ectoderm (Fig. 1b). Totipotency of ESC-like colonies was further validated by analyzing their ability to form teratomas in NOD/SCID mice. Accordingly, NOD/SCID mice were subcutaneously/intramuscularly injected with ESC-like colonies and the formation of teratomas was scrutinized. Sections of teratoma stained with hematoxylin and eosin confirmed the presence of all three germ layers formed by the differentiation of ESC-like colonies: gut epithelium accounted for endoderm; cartilage for mesoderm; and neural rosettes and retinal pigment for ectoderm (Fig. 1c). ESC-like colonies expressed the iPSC markers OCT4, NANOG, TRA-1-81, TRA-1-60, and SSEA4 (Fig. 1d). Taken together, these results confirm the identity of ESC-like colonies induced from urinary cells as iPSCs.

### Differentiation of iPSCs into otic progenitors, hair cell-like cells, and SGN-like cells

**Fig. 2:**
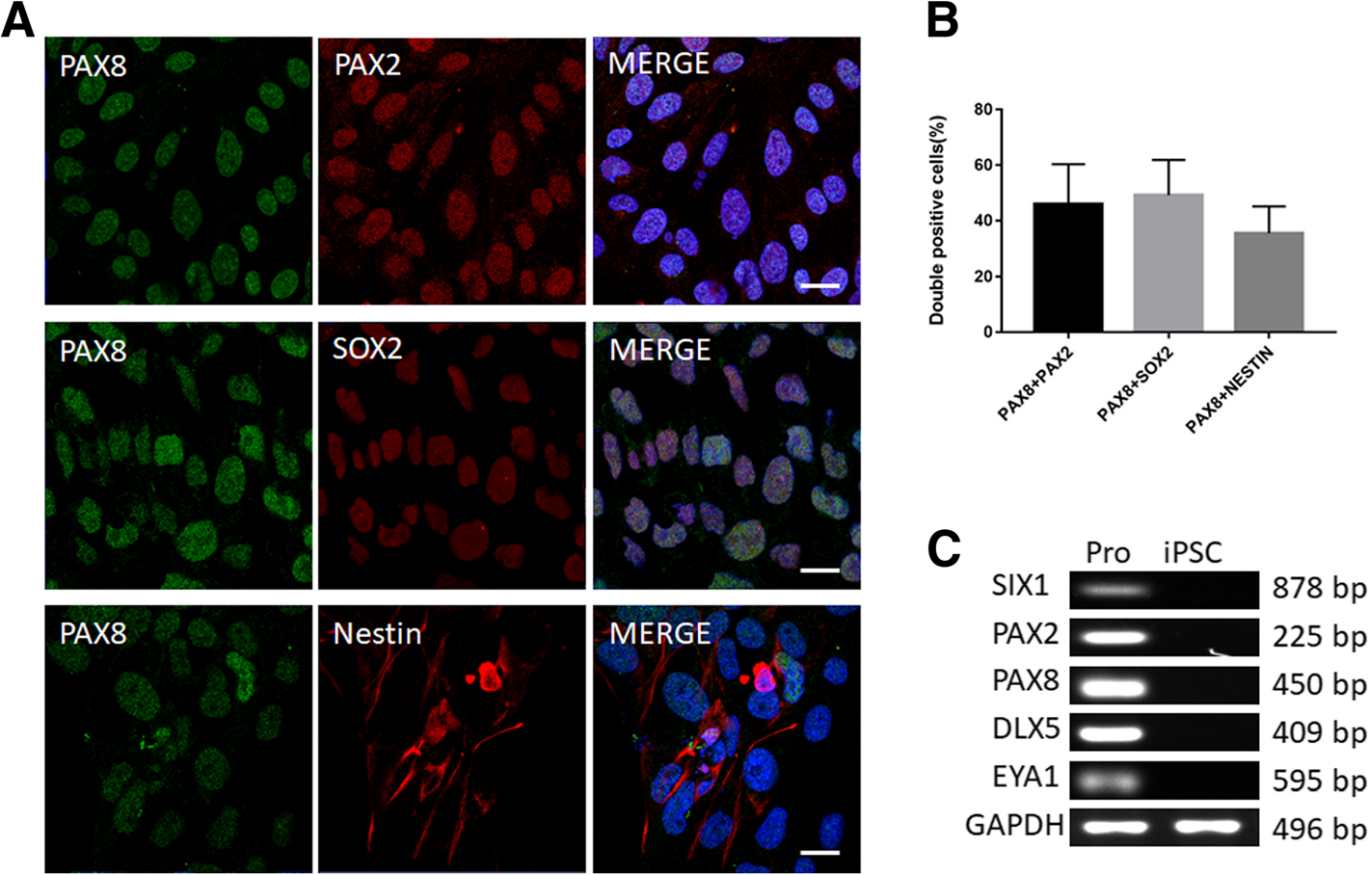
Induction of induced pluripotent stem cells (iPSCs) to differentiate into otic progenitors. **a** iPSCs were induced to differentiate into otic progenitors and were stained for the presence of early otic markers PAX8 and PAX2, PAX8 and SOX2, and PAX8 and Nestin. Nuclei were stained with 4′,6-diamidino-2-phenylindole (DAPI; blue). Scale bars = 20 μm. **b** Percentage of double-positive cells 12 days postinduction for PAX8 and PAX2, PAX8 and SOX2, and PAX8 and Nestin (*n* = 6, mean ± SD). **c** Reverse transcription-polymerase chain reaction (RT-PCR) analysis of gene expression of early otic markers SIX1, PAX2, PAX8, DLX5, and EYA1. The housekeeping gene glyceraldehyde-3-phosphate dehydrogenase (GAPDH) was used as an internal reference

iPSCs were induced to differentiate into otic progenitors using the otic inducing factors FGF3 and FGF10. At 12 days postinduction, two morphologically distinct types of otic progenitor colonies (OEPs and ONPs) were formed. OEPs and ONPs are progenitors of hair cells and SGNs, respectively [16]. Immunoreactive staining detected the enhanced expression of PAX8, PAX2, and SOX2 (otic progenitor makers) in otic progenitor colonies (Fig. 2a). Expression of Nestin (ONP marker) in the cytoplasm of ONPs and its absence in OEPs warrants its use in distinguishing between ONPs and OEPs. Double-labeled staining for PAX8 and Nestin confirmed the specificity of Nestin in ONPs (Fig. 2a). Our results suggest percentage coexpression of 46.0 ± 13.1% (*n* = 6) of cells coexpressing PAX8 and PAX2, 49.2 ± 11.6% (*n* = 6) of cells coexpressing PAX8 and SOX2, and 35.5 ± 8.8% (*n* = 6) of cells coexpressing PAX8 and Nestin, indicating the efficient generation of otic progenitor cells from iPSCs (Fig. 2b). Gene expression analysis revealed the expression of early placodal markers SIX1, PAX8, PAX2, DLX5, and EYA1 induced by combined treatment with FGF3 and FGF10 (Fig. 2c). Thus, these results confirm the ability of iPSCs derived from urinary cells to differentiate into otic progenitors.

**Fig. 3:**
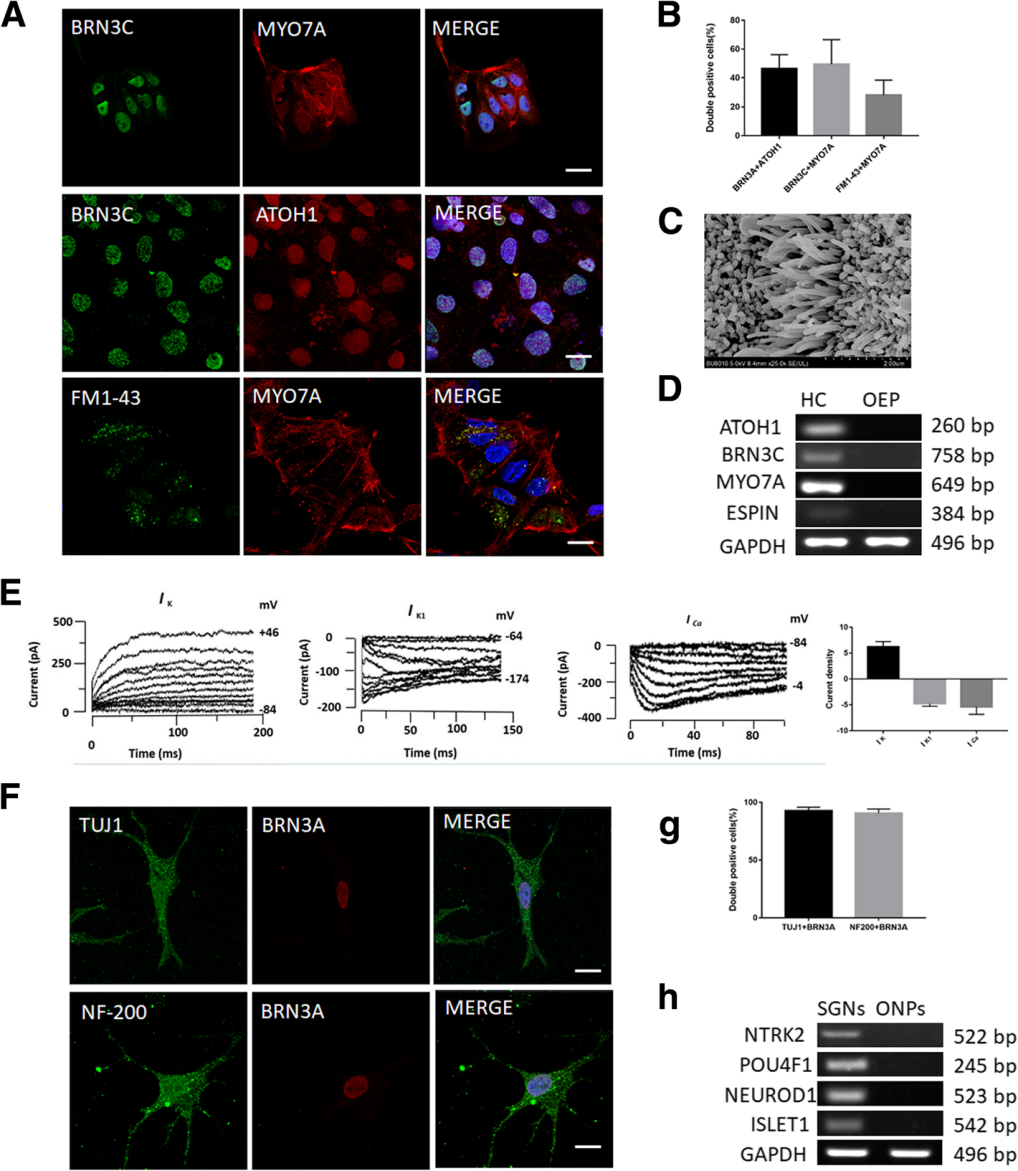
Differentiation of otic progenitors into hair cell-like cells and spiral ganglion neuron (SGN)-like cells. **a** Cells derived from otic epithelial progenitors (OEPs) expressed hair cell markers BRN3C, MYO7A, and ATOH1. Uptake of FM1–43 was detected. Scale bars = 20 μm. **b** Percentage of double-positive cells: 49.42 ± 15.49% of cells expressing both BRN3C and MYO7A, 46.42 ± 8.9% of cells positive for BRN3C and ATOH1, and 28.26 ± 9.2% of cells double-labeled with FM1–43 and MYO7A (*n* = 6, mean ± SD). **c** After 3 weeks of culture on mitotically inactivated chicken utricle stromal cells, the appearance of apical projections reminiscent of a hair bundle on the surface of induced cells was detected by scanning electron microscopy (SEM). Scale bar = 1.0 μm. **d** Upregulated expressions of hair cell markers ATOH1, BRN3C, MYO7A, and ESPIN detected by gene expression analysis in respective cells compared to that in OEPs. **e** Outward K^+^ (*I*_K_), inward K^+^ (*I*_K1_) and inward Ca^2+^ (*I*_ca_) currents recorded from induced hair cell-like cells. Currents were elicited by 10 voltage steps from the holding potential (left and right panel: −84 mV; middle panel: −64 mV). Peak current voltages are also indicated. The current density (pA/pF) of *I*_K_, *I*_K1_, and *I*_Ca_ was 6.22 ± 0.90, −4.78 ± 0.44, and −5.40 ± 1.29, respectively (*n* = 8, mean ± SD). **f** SGN-like cells derived from otic neural progenitors (ONPs) were positive for NF200, β-Tubulin III (TUJ1), and BRN3A (POU4F1). **g** Percentage of double-positive cells: 92.8 ± 2.6% and 90.79 ± 3.0% of induced cells were double positive for TUJ1 and BRN3A, and NF200 and BRN3A, respectively (*n* = 4, mean ± SD). **h** Expression of neuronal markers NTRK2, BRN3A, NEUROD1, and ISLET1 detected by reverse transcription-polymerase chain reaction (RT-PCR). The housekeeping gene glyceraldehyde-3-phosphate dehydrogenase (GAPDH) was used as an internal reference

OEPs enriched from otic progenitors were plated on mitotically inactivated chicken utricle stromal cells to induce differentiation into hair cells. At 20 days postinduction, an immunocytochemistry assay was performed to test for the expression of markers specific for hair cells. Immunoreactive staining was positive for BRN3C and MYO7A in induced cells and nuclear immunoreactivity was detected for BRN3C and ATOH1 (Fig. 3a). Percentage coexpression of BRN3C and MYO7A was 49.42 ± 15.49%, and that of BRN3C and ATOH1 was 46.42 ± 8.9% (Fig. 3b). Previous reports suggest rapid entry of FM1–43 dye into hair cells via mechanotransduction channels [17, 18]. Immunoreactive staining detected double-labeled hair cells with FM1–43FX and MYO7A (28.2 ± 9.2%), indicating the presence of potentially functional mechanotransduction channels in induced hair cell-like cells (Fig. 3a, b). Mechanosensory stereocilia bundles present on the surface of hair cells play an important role in auditory signal transduction [19, 20]. In this study, few induced cells developed apical projections that are reminiscent of hair bundles as seen in SEM (Fig. 3c). Gene expression analysis revealed the expression of hair cell markers ATOH1, BRN3C, MYO7A, and ESPIN by hair cell-like cells (Fig. 3d). Electrophysiological analysis using the patch clamp technique detected a voltage-dependent outward K^+^ current (*I*_K_), an inward K^+^ current (*I*_K1_), and an inward Ca^2+^ current (*I*_Ca_) in induced hair cell-like cells attributing electrophysiological properties to these cells (Fig. 3e). Current densities of *I*_K_, *I*_K1_, and *I*_Ca_ were 6.22 ± 0.90, −4.78 ± 0.44, and −5.40 ± 1.29, respectively (Fig. 3e). Thus, OEPs derived from iPSCs are capable of differentiating into hair cell-like cells possessing significant characteristics of hair cells.

ONPs enriched from otic progenitors were cultured under neutralizing conditions. At 12 days postinduction, induced cells showed protrusions typical of dendrites seen in sensory neurons (Fig. 3f). Immunoreactive staining indicated the induced cells to be positive for both BRN3A and β-tubulin III (TUJ1), and BRN3A and NF200, wherein three of these markers were specific for sensory neurons (Fig. 3f). Coexpression of TUJ1 and BRN3A was 92.84 ± 2.58%, and of NF200 and BRN3A was 90.79 ± 2.95% (Fig. 3g). RT-PCR revealed expression of the neuronal markers NTRK2 (TrkB), POU4F1 (BRN3A), NEUROD1, and ISLET1 by induced cells (Fig. 3h). These results demonstrate the possibility of inducing iPSC-derived ONPs to differentiate into SGN-like cells.

### Synaptic connection between hair cell-like cells and SGNs

**Fig. 4:**
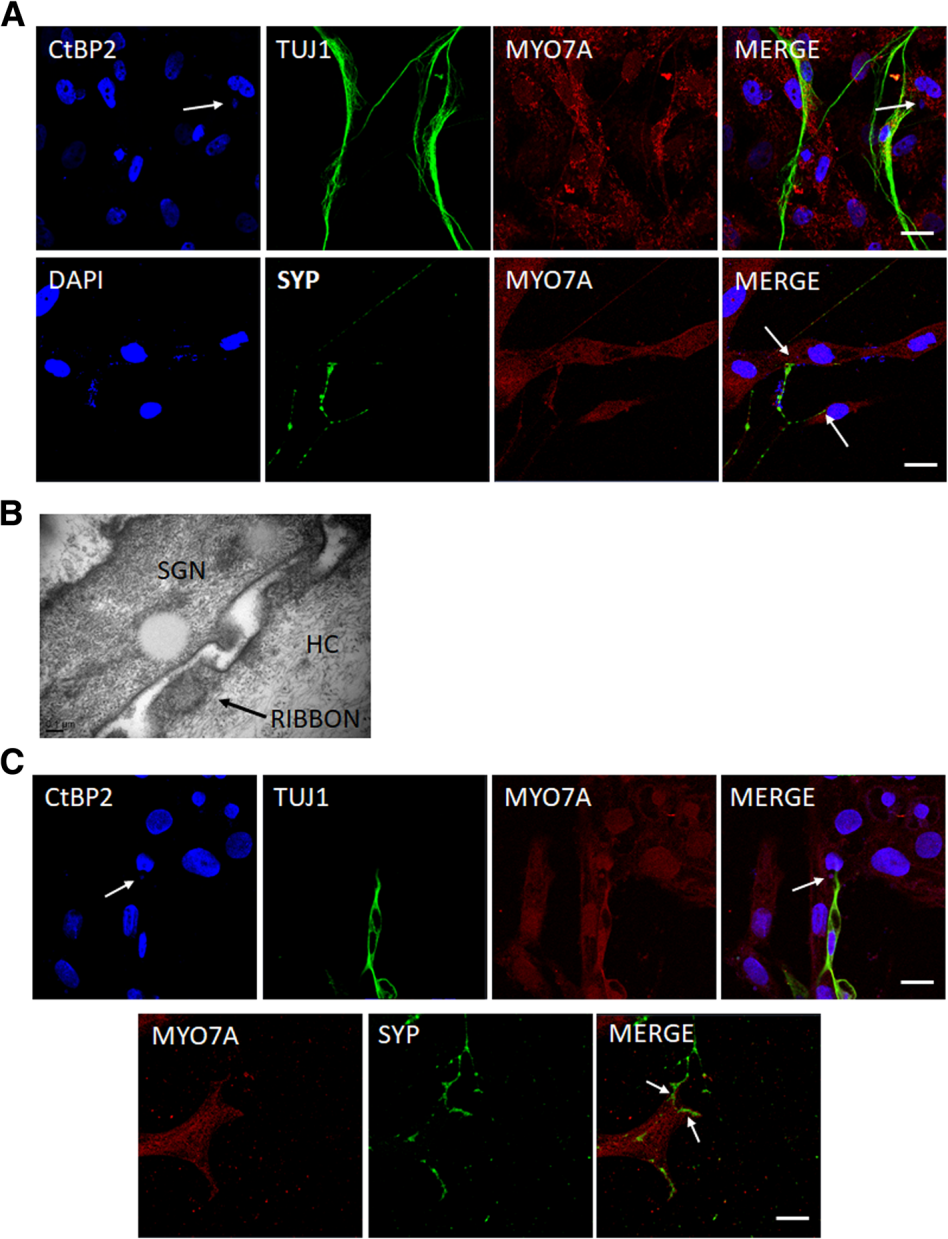
Synaptic connections between hair cell-like cells and spiral ganglion neurons (SGNs) or SGN-like cells. **a** In coculture, SGNs extended their neural dendrites towards hair cell-like cells and expressed the synaptic connection markers C-terminal binding protein 2 (CtBP2) in the cytoplasm of hair cell-like cells (arrow) and synaptophysin (SYP) in the SGN (arrow). Scale bars = 20 μm. **b** The structure resembling ribbon in hair cells observed by transmission electronic microscopy (TEM) (arrow). Scale bar = 0.1 μm. **c** In coculture, CtBP2 in hair cell-like cells (arrow) and SYP (arrow) in SGN-like cells detected by immunoreactive staining. Scale bars = 20 μm

In cochlea, hair cells are connected with SGNs facilitating the conduction of signals between them. To examine the presence of this functional connection between induced hair cell-like cells and SGNs, induced hair cell-like cells were cocultured with SGNs harvested from neonatal mice. After 7 days of coculture, neurons expressing TUJ1 projected their neurites towards induced hair cell-like cells expressing MYO7A. Few processes of the SGNs were branched and the branches showed well-defined directional growth towards individual hair cells (Fig. 4a). These results demonstrate the ability of mouse SGNs to project their neurites towards hair cell-like cells. C-terminal binding protein 2 (CtBP2) is a principal component of ribbon protein [21] and plays an important role in enabling synchronous auditory signaling. It is expressed both ubiquitously in the nucleus of hair cell-like cells and specifically in the cytoplasm of hair cell-like cells stretched with the nerve endings of SGNs [22]. In cocultured cells, CtBP2 was detected in the nuclei and in the cytoplasm of hair cell-like cells adjacent to nerve endings. Few CtBP2-positive puncta were identified in the cytoplasm (Fig. 4a). Formation of synapses between induced hair cell-like cells and SGNs was further investigated by analyzing the expression of the synaptic protein synaptophysin (SYP), an intrinsic membrane protein of small synaptic vesicles. In cocultured cells, SYP was expressed at the nerve endings of SGNs adjacent to hair cell-like cells (Fig. 4a). TEM detected structures resembling ribbon in hair cells (Fig. 4b). These findings indicate the formation of active synapses between iPSC-derived hair cell-like cells and SGNs.

Hair cell-like cells and SGN-like cells were cocultured and examined for the formation of synaptic connections between iPSC-derived hair cells and iPSC-derived SGN-like cells. After 7 days of coculture, the neurites of TUJ1-positive SGN-like cells stretched towards induced hair cell-like cells. Immunocytochemistry analysis detected CtBP2 in the cytoplasm of hair cell-like cells adjacent to the nerve endings (Fig. 4c). Immunoreactive staining detected expression of SYP in SGN-like cells connected with MYO7A-positive hair cell-like cells (Fig. 4c). Immunoreactive staining confirmed the formation of a synaptic connection between hair cell-like cells and SGN-like cells in vitro.

### Transplantation of otic epithelial progenitors into the cochlea of mouse

**Fig. 5:**
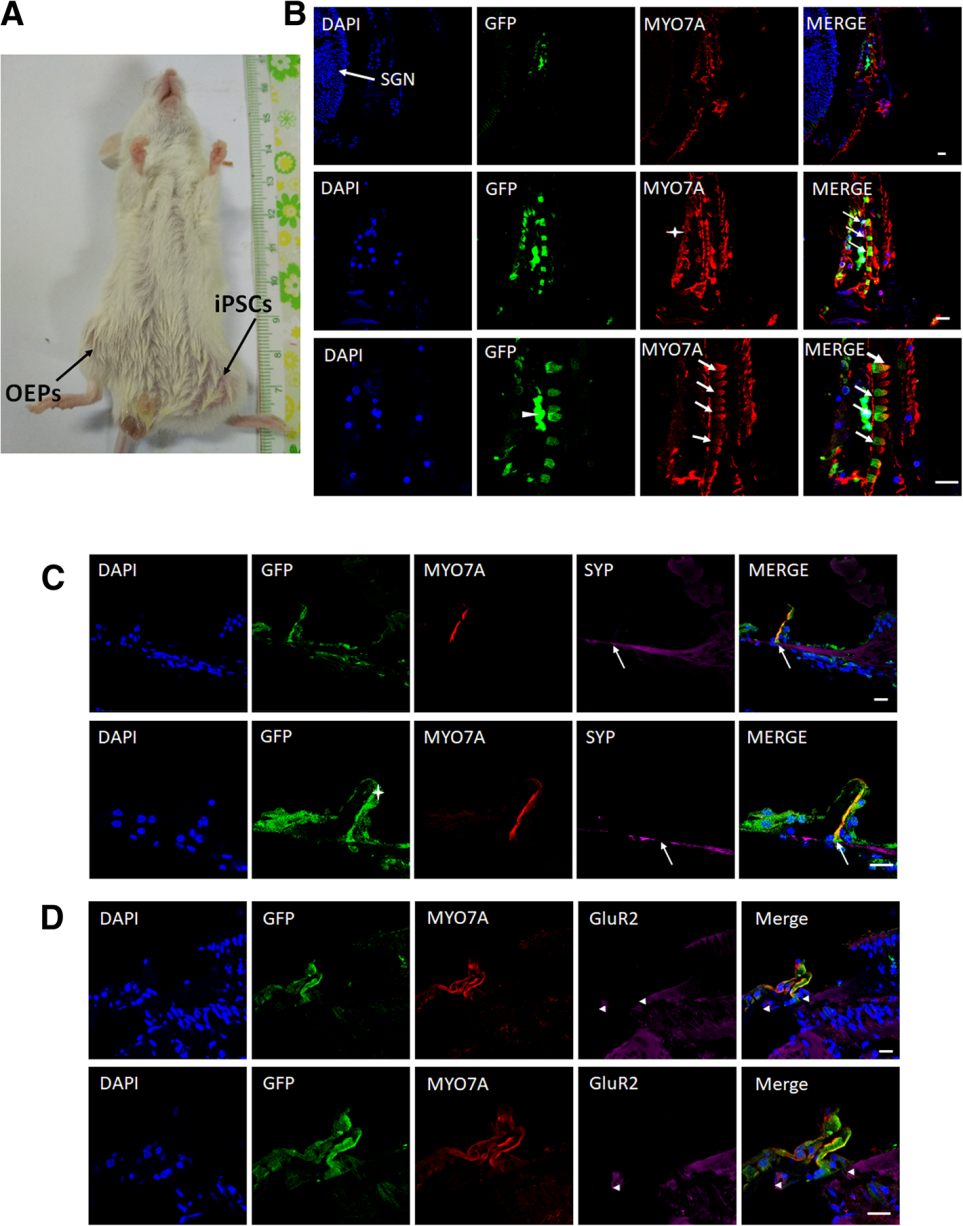
Transplantation of otic epithelial progenitors into the cochlea of the mouse. **a** The left leg of NOD/SCID mouse injected with induced pluripotent stem cells (iPSCs) formed an obvious bulge, while the right leg injected with otic epithelial progenitors (OEPs) was normal. **b** Location of transplanted cells in the organ of Corti corresponding to the location of native spiral ganglion neurons (SGNs) (arrow). The first row of figures indicates migration of few MYO7A-positive transplanted cells to the site of resident hair cells. The second row of figures indicates the location of MYO7A-positive transplanted cells in the greater epithelial ridge (asterisk) and inner hair cell row (arrows). The third row of figures indicates migration of few cells to the site of missing inner hair cells and their differentiation into hair cell-like cells (arrows). Few transplanted cells did not differentiate into hair cells (arrowhead). **c** Fibers of native SGNs approaching the hair cells regenerated from transplanted cells expressing synaptophysin (SYP) (arrows). Location of differentiated cells in the organ of Corti (asterisk). **d** Native SGNs targeted cells migrating into the organ of Corti, and fibers positive for GluR2 lay adjacent to the basal pole of green fluorescent protein (GFP)-positive cells (arrowhead). All scale bars = 20 μm

Prior to the transplantation experiment, NOD/SCID mice were subcutaneously/intramuscularly injected with OEPs at a density of 1 × 10^7^ cells to detect the tumorigenic capacity of transplanted cells in vivo. After 3 months, palpable tumors appeared in the skin of mice injected with iPSCs compared with those injected with OEPs where tumorigenesis was not detected (Fig. 5a). These results indicate the inability of iPSC-derived OEPs to form tumors compared with the tumorigenic iPSCs and indicate they should therefore be a secure candidate for transplantation into the cochlea.

Subsequently, OEPs derived from iPSCs expressing GFP were transplanted into *Slc26a4*-null mice to examine the potential of engrafting and differentiation of transplanted cells in the organ of Corti of the mouse. After 4 weeks of transplantation, based on the location of native hair cells and SGNs examined by cochlea basilar membrane stretched preparation technique, some MYO7A-positive transplanted cells were found to be integrated at the site where inner hair cells were lost (Fig. 5b), and other MYO7A-expressing transplanted cells were located in the region of the greater epithelial ridge. However, few of the transplanted cells that migrated into the organ of Corti could not differentiate into hair cells. Compared with the stretched preparation, frozen sections were able to reflect the location and the direction of migration of transplanted cells. We found that a small fraction of MYO7A-negative cells were located in the scala tympani (Fig. 5c). Scala tympani connect with the round window membrane and are the place where the transplanted cells first arrive. Some of the transplanted cells migrated through the basilar or Reissner’s membrane into the scala media. Consistent with the above findings by stretched preparation, thin cochlear sections revealed the inability of transplanted cells located in the endolymphatic space to differentiate into hair cells, and only a part of cells located in the organ of Corti could differentiate into hair cells. However, transplanted cells migrating into the organ of Corti, although positive for MYO7A, failed to arrange regularly as native hair cells. Immunoreactive staining of SYP was used to detect SGNs underneath GFP-positive cells labeled with MYO7A (Fig. 5c). Native SGNs targeted the transplanted cells located in the organ of Corti, and fibers positive for GluR2 were located adjacent to the basal pole of GFP-positive cells (Fig. 5d). Taken together, these results indicate the ability of transplanted cells to migrate, engraft, and differentiate into hair cells, and the ability of transplanted cells located in the organ of Corti to form synaptic connections with native SGNs.

## Discussion

SNHL is a common form of sensory impairment resulting from the loss of inner hair cells or SGNs. Unfortunately, mammalian cochlear sensory hair cells fail to regenerate and therefore hearing loss resulting from the death of hair cells is irreversible [23]. Over several years, researchers have tried various strategies to regenerate inner ear hair cells, including re-expression of Atonal homolog1 (Atoh1) [24], interference of Notch [25–27], deletion of targeted p27Kip1 [28], exogenous activation of canonical WNT signaling [29, 30], and various gene therapies [31, 32]. In a recent study, progressive hearing loss seen in the Tmc1Beethoven (Bth) mouse model was substantially alleviated by injecting Cas9–guide RNA–lipid complexes targeting the Tmc1^Bth^ allele into the cochlea of neonatal Tmc1^Bth/+^ mice [33]. Stem cell transplantation into the cochlea using neural stem cells [34] and miPSCs [35, 36] is a relatively new strategy for rescuing hearing loss. In vitro, hESC-derived otic progenitors could differentiate into hair cell-like cells and sensory neurons. Furthermore, transplantation of otic neural progenitors into the cochlea significantly improved the auditory-evoked response thresholds [16].

As iPSC lines can be established by reprograming somatic cells to ESC-like cells using the four transcription factor genes (OCT-4, SOX-2, KLF-4, and c-MYC) [4], iPSC research has attracted great attention for use in the clinic. iPSCs induced from somatic cells of patients with hereditary diseases and cells differentiated from iPSCs have been used for disease models to study the molecular mechanisms of hereditary etiopathology. Recently, human causative genes in iPSCs derived from patients with hereditary hearing loss were genetically corrected using the CRISPR/Cas9 system to restore the morphology and functioning of hair cell-like cells derived from these iPSCs [3, 9]. Therefore, somatic cell reprogramming technology offers unprecedented opportunities for future clinical applications. In this study, we established an iPSC line by reprogramming urinary cells isolated from a normal donor. We examined the ability of hair cell-like cells derived from iPSCs to form synaptic connections with SGNs. OEPs derived from iPSCs were transplanted into the cochlea of the mouse to examine engrafting and differentiation of transplanted cells committed to form hair cells in the organ of Corti.

For the differentiation of otic progenitors and hair cell-like cells from ESCs or iPSCs in vitro, several induction protocols were developed, including single-layer induction, three-dimensional aggregate culture, and EB induction combined with single-layer induction [16, 37, 38]. In our study, the single-layer induction protocol was used to induce the differentiation of iPSCs into the otic progenitors ONPs and OEPs, capable of forming SGN-like cells and hair cell-like cells, respectively. Hair cell-like cells expressed marker genes specific for the inner ear hair cell, including MYO7A, BRN3A, and ATHO1. Perception of sound by the ear depends on the functioning of specialized sensory epithelial hair cells equipped with microvillus-like cellular projections called stereocilia [39]. In our study, protrusions seen on the surface of hair cell-like cells were reminiscent of stereocilia in hair cells, suggesting that induced hair cell-like cells might possess the structure required for sound detection. Previous studies reported rapid entry of styryl dye FM1–43 into the cytosol of hair cells via mechanotransducer channels [18, 40]. Hair cells detect vibrations of their stereociliary bundle through the activation of mechanically-sensitive transducer (MET) channels and convert mechanical signals into electrical impulses [41]. Two-photon imaging of FM1–43 indicated the existence of mechanotransducer channels in hair cell-like cells. A principal step in the initiation of signal propagation to afferent neurons is the driving force required for the entry of K^+^ and Ca^2+^ [42]. In our study, *I*_K_, *I*_K1_, and *I*_ca_ were detected in hair cell-like cells using an electrophysiological technique. The normal current density of *I*_K_, *I*_K1_, and *I*_ca_ detected in hair cell-like cells implied a normal physiological activity. In summary, our results revealed that induced hair cell-like cells possessed characteristics of hair cells.

Previous studies reported the formation of synaptic connections between ESC-derived neurons and inner hair cells, and that native SGNs could extend their neurites and innervate ectopic hair cell-like cells induced by overexpression of Atoh1 in vitro [43, 44]. However, the synapse-forming potential of iPSC-derived hair cell-like cells with SGNs or iPSC-derived sensory neurons has not been elucidated. We examined the ability of iPSC-derived hair cell-like cells to form synaptic connections with SGNs or iPSC-derived sensory neurons. In this study, afferent synaptogenesis between hair cell-like cells and SGNs was observed by a series of immunoreactive stainings. The synaptic ribbon is an electron-dense structure associated with presynaptic active zones at sensory synapses in the inner ear. It serves as a conveyor belt that maintains vesicles and traffics vesicles to release sites during the release of the neurotransmitter [45, 46]. CtBP2/RIBEYE is the major protein that forms the ribbon scaffold [47]. Knockout (KO) mice lacking RIBEYE had a mild hearing deficit [15, 48]. In the present study, hair cell-like cells adjacent to SGNs showed presynaptic punctate expression of CtBP2. Expression of SYP, a membrane protein specific to synaptic vesicles, correlates directly with the presence of neurotransmitter [49]. Expression of SYP was detected at the nerve endings of SGNs adjoining hair cell-like cells. Therefore, the presence of CtBP2-positive puncta and a SYP-positive expression indicated the establishment of afferent synaptic connections between hair cell-like cells and SGNs. Formation of cochlear afferent synapses between hair cell-like cells and SGNs might provide an important foundation for regenerative approaches aimed at restoring hearing after hair cell loss.

Furthermore, OEPs derived from iPSCs were transplanted into the cochlea of the mouse to examine engrafting and differentiation of transplanted cells committed to form hair cells in the organ of Corti. Previous studies reported the possible association of iPSCs with a high risk of tumor formation in the cochlea [50]. In the present study, we used OEPs derived from iPSCs expressing GFP as a secure candidate for transplantation in the cochlea. The tumor-forming risk of these progenitors was evaluated before transplantation. In vivo, compared with iPSCs, OEPs derived from iPSCs did not form tumors, confirming the loss of their tumor-forming potential and therefore their safety for cell delivery. OEPs derived from iPSCs were transplanted into the cochlea of *Slc26a4-*null mice (exhibiting complete loss of hearing) through the round window membrane. After 4 weeks of transplantation, iPSC-derived OEPs migrated past the membrane that quarantined the perilymph and endolymph. Some of the transplanted cells migrated into the scala media where hair cells were present. A fraction of GFP-positive cells remained at the scala tympani, and some of the transplanted cells could migrate into the scala media but failed to transform into hair cells or to migrate to the right site. Remarkably, some cells that integrated at the site of resident hair cells were capable of differentiating into hair cells. SYP, an abundant integral membrane protein of synaptic vesicles, plays a significant role in the regulation of neurotransmitter release, synaptic plasticity, and participates in the recycling of synaptic vesicles [51, 52]. GluR2, an AMPA receptor, is associated with synaptic transmission and synaptic plasticity [53]. In this study, MYO7A-positive transplanted cells were able to establish synaptic connections with native SGNs, suggesting the establishment of synaptic transmission. However, only a few of the transplanted cells were integrated at the site where hair cells were lost and, although these cells could differentiate into cells expressing MYO7A and form synaptic connection with native SGNs, this may be not enough for recovery of hearing. Thus, some modifications are warranted for future research. Firstly, to increase the number of cells reaching the scala media, a part of the perilymph could be suctioned out before transplantation to increase the available space for transplanting more cells. Alternatively, larger animals such as guinea pigs could be considered for use as animal models. Secondly, to facilitate integration of transplanted cells into the organ of Corti, sodium caprate could be used for transient disruption of auditory epithelium junctions, endolymph could be replaced with perilymph, and stria vascularis pumps could be blocked with furosemide [54]. Finally, compared with the induction of otic progenitors and differentiation of hair cell-like cells as monolayers [16], the differentiation protocol of EBs and three-dimensional aggregates did not depend on mitotically inactivated chicken feeders and could simulate internal environment [37, 38]. Therefore, the differentiation protocol of EBs and three-dimensional aggregates may be useful for the differentiation of otic progenitors into hair cells in cochlea of the mouse and enhance cell engraftment and integration.

In summary, the present study confirmed the ability of iPSC-derived OEPs to differentiate into hair cell-like cells exhibiting characteristics of hair cells. As a novelty in this research, OEP-derived hair cell-like cells were examined the formation of synaptic connections with SGNs in vitro and in vivo, and showed that the hair cell-like cells had the potential for synaptic transmission. In addition, the transplanted OEPs could migrate into the organ of Corti and subsequently expressed hair cell markers, and the differentiated cells could form synaptic connections with native SGNs. Integration and differentiation of iPSC-derived OEPs in cochlea demonstrated the feasibility for regenerating hair cells in vivo. However, further studies are needed to explore ways to improve the auditory function through stem cell transplantation.

## Conclusion

In the present study, urinary cells were reprogramed into iPSCs that were induced to differentiate into otic progenitors, hair cell-like cells, and SGN-like cells. In vitro, hair cell-like cells derived from OEPs formed synaptic connections with SGNs dissected from the mouse or SGN-like cells derived from ONPs. On the premise of secure cell transplantation, OEPs derived from iPSCs were delivered into the cochlea. A few of the OEPs migrated into the organ of Corti and differentiated into hair cells. The dendritic endings of native SGNs formed synaptic connections with adjacent hair cells regenerated from the transplanted cells. These results offer substantial promise for a cell-based therapy for hearing damage resulting from loss of hair cells.
